# Atypical Rash and Sequential Bilateral Bell’s Palsy in a Patient with Lyme Disease: A Case Report

**DOI:** 10.51894/001c.144457

**Published:** 2025-09-30

**Authors:** Marrem Gago

**Affiliations:** 1 College of Osteopathic Medicine Michigan State University

## 79

### BACKGROUND

Lyme disease, caused by Borrelia burgdorferi, is a tick-borne illness that progresses through distinct stages, often presenting with erythema migrans, flu-like symptoms, and potential neurological complications. While unilateral Bell’s palsy is a known manifestation, sequential bilateral Bell’s palsy is exceedingly rare, with an incidence of 1 in 5,000,000 cases. Misdiagnosis can delay treatment, increasing the risk of complications.

### CASE PRESENTATION

A 44-year-old female presented with flu-like symptoms and a uniform erythematous rash, initially diagnosed as cellulitis and treated with Cephalexin. Over the next month, she developed right-sided Bell’s palsy, followed by left arm weakness. After receiving a five-day course of Prednisone, her right-sided palsy partially improved, but left-sided Bell’s palsy developed two weeks later. Lyme serology was positive (index 7.49, reactive IgG/IgM bands). Treatment with intravenous Ceftriaxone (2g daily) was initiated 57 days after rash onset, leading to near-complete neurological recovery within one day.

### DISCUSSION

This case underscores the diagnostic challenges of Lyme disease, particularly when early signs mimic cellulitis. The delayed initiation of appropriate antibiotics likely contributed to progressive neurological involvement. While Bell’s palsy is common in Lyme disease, bilateral sequential involvement is rare. Additionally, the use of corticosteroids in Lyme-associated Bell’s palsy remains controversial, with some studies suggesting worsened long-term outcomes. This case supports avoiding corticosteroids in suspected Lyme neuroborreliosis and highlights the importance of early antibiotic therapy.

### CONCLUSION

This case highlights the importance of considering Lyme disease in patients with nonspecific rashes and neurological symptoms. The atypical rash and sequential Bell’s palsy emphasize the potential for misdiagnosis and delayed treatment. Early recognition, serologic testing, and appropriate antibiotic therapy are crucial in preventing long-term complications. Additionally, this case contributes to the discussion on the role of corticosteroids in Lyme disease, reinforcing the need for caution when prescribing them in Lyme-associated facial palsy.

